# Review of Neurological Manifestations of SARS-CoV-2

**DOI:** 10.7759/cureus.38194

**Published:** 2023-04-27

**Authors:** Priyal ., Vineet Sehgal, Saniya Kapila, Rishabh Taneja, Prachi Mehmi, Nihal Gulati

**Affiliations:** 1 Neurology, Indraprastha Apollo Hospitals, New Delhi, IND; 2 Neurology, Sehgal’s Neuro & Child Care Center, Amritsar, IND; 3 General Practice, Fortis Escorts Hospital, Amritsar, IND; 4 Medicine, Government Multi-Specialty Hospital, Chandigarh, IND; 5 Graduate Medical Education, Adesh Institute of Medical Sciences & Research, Bathinda, IND; 6 Neurology, Adesh Institute of Medical Sciences & Research, Bathinda, IND; 7 General Practice, Navpreet Hospital, Amritsar, IND

**Keywords:** covid-19 neurological complications, correlation and association research, clinicopathological correlation, anatomo-radiological correlation, neurotropic virus

## Abstract

Severe acute respiratory syndrome coronavirus 2 (SARS-CoV-2) can affect any part of the neuraxis. Many neurological conditions have been attributed to be caused by SARS-CoV-2, namely encephalopathy (acute necrotizing encephalopathy and encephalopathy with reversible splenial lesions), seizures, stroke, cranial nerve palsies, meningoencephalitis, acute disseminated encephalomyelitis (ADEM), transverse myelitis (long and short segment), Guillain-Barré syndrome (GBS) and its variants, polyneuritis cranialis, optic neuritis (ON), plexopathy, myasthenia gravis (MG), and myositis. The pathophysiology differs depending on the time frame of presentation. In patients with concomitant pulmonary disease, for instance, acute neurological illness appears to be caused by endotheliopathy and cytokine storm. Autoimmunity and molecular mimicry are causative for post-coronavirus disease 2019 (COVID-19)-sequelae. It has not yet been shown that the virus can penetrate the central nervous system (CNS) directly. This review aims to describe the disease and root pathogenic cause of the various neurological manifestations of COVID-19. We searched Pubmed/Medline and Google Scholar using the keywords “SARS-CoV-2” and “neurological illness” for articles published between January 2020 and November 2022. Then, we used the SWIFT-Review (Sciome LLC, North Carolina, United States), a text-mining workbench for systematic review, to classify the 1383 articles into MeSH hierarchical tree codes for articles on various parts of the nervous system, such as the CNS, peripheral nervous system, autonomic nervous system, neuromuscular junction, sensory system, and musculoskeletal system. Finally, we reviewed 152 articles in full text. SARS-CoV-2 RNA has been found in multiple brain areas without any histopathological changes. Despite the absence of in vivo virions or virus-infected cells, CNS inflammation has been reported, especially in the olfactory bulb and brain stem. SARS-CoV-2 genomes and proteins have been found in affected individuals’ brain tissues, but corresponding neuropathologic changes are seldom found in these cases. Additionally, viral RNA can rarely be identified in neurological patients’ CSF post hoc SARS-CoV-2 infection. Most patients with neurological symptoms do not have active viral replication in the nervous system and infrequently have typical clinical and laboratory characteristics of viral CNS infections. Endotheliopathy and the systemic inflammatory response to SARS-CoV-2 infection play a crucial role in developing neuro-COVID-19, with proinflammatory cytokine release mediating both pathological pathways. The systemic inflammatory mediators likely activate astrocytes and microglia across the blood-brain barrier, indirectly affecting CNS-specific immune activation and tissue injury. The management differs according to co-morbidities and the neurological disorder.

## Introduction and background

Coronavirus disease 2019 (COVID-19) causes flu-like illness in the majority of people. Comorbidities such as hypertension, diabetes, or lung and cardiac disease increase the severity and risk of death in the elderly. In critically ill individuals with comorbidities, neurological problems are frequently present. COVID-19 can impact the central nervous system (CNS) and peripheral neural systems (PNS). Neurological illness can be a presenting feature of severe acute respiratory syndrome coronavirus 2 (SARS-CoV-2) infection, occur together with respiratory illness, or develop after recovery from respiratory illness. In patients with concurrent pulmonary disease, for example, acute neurological illness appears to be triggered by endotheliopathy and cytokine storm [1]. A typical cytokine storm can affect the CNS milieu by causing significant metabolic alterations and organ failure. The hypoxic environment generates severe neurological signs, such as altered sensorium, agitation, swidelirium, and coma. Severe coagulopathies can cause ischemic or hemorrhagic stroke. Autoimmunity and molecular mimicry cause post-COVID-19 sequelae. In a retrospective study performed on COVID-19 patients in Wuhan, China, neurological symptoms were observed in 36.4% of the total patients and 45.5% of patients suffering from severe COVID-19 [2]. A nonspecific headache is the most prevalent neurological complaint, ranging from 1.7% to 33.9%. A novel type of headache has been identified as a “personal protection equipment-related headache" seen in first-line healthcare workers. Anosmia, whether complete or partial and ageusia are common PNS manifestations. Myalgia and tiredness are prevalent features with a combined rate range of 1.5% to 61%, and increased creatine kinase values indicate muscular damage [3-4]. Guillain Barré syndrome (GBS) in COVID-19 individuals has recently been identified, and a postinfectious immune-mediated inflammatory process seems to be the underlying pathogenesis responsive to intravenous immunoglobulin (IVIG) and plasmapheresis.

We conducted our review using the PubMed/MEDLINE (Medical Literature Analysis and Retrieval System Online, or MEDLARS Online) and Google Scholar databases from January 2020 to November 2022, searching for the key terms “SARS-CoV-2” and “neurological disease.” We applied the boolean automation tools “AND,” “NOT,” and “OR” wherever applicable. In addition, we used search filters such as studies done on the human population and reported in the English language. The search results gave 1383 articles. We could categorize all 1383 articles into the Medical Subject Headings (MeSH) hierarchical tree codes for the nervous system with the help of the SWIFT (Sciome Workbench for Interactive computer-Facilitated Text-mining)-Review (Sciome LLC, North Carolina, United States), a text-mining workbench for systematic review, for articles on various parts of the nervous system such as the CNS, PNS, autonomic nervous system (ANS), neuromuscular junction, sensory system, and musculoskeletal system. Finally, we reviewed 152 articles in full text.

## Review

CNS and COVID-19

Infectious exosomes containing SARS-CoV-2 are formed and trafficked across the lungs and the brain via intravascular compartment and the SARS-CoV-2 virus can affect any part of the neuraxis [3-4]. Neurological complications depend on the time frame of presentation with respect to COVID-19 infection, the severity of respiratory illness, and the type of COVID-19 vaccine administered in a few neurological cases. The neurological symptoms may come on suddenly or develop gradually over time. The neurological manifestation of SARS-CoV-2 can occur via various mechanisms; The direct viral infection of the tissues (equivocal), the development of autoimmunity, molecular mimicry in which para-infectious antibodies against neural tissues or vascular endothelium are formed, and via endotheliopathy of the sentinel brain microvascular endothelial cell (BMECs), which can indirectly manifest as a neurological disease and is antecedent to the formation of para-infectious antibodies [4-7]. Finally, long-COVID-19 can manifest in either a relapsing-remitting form or a continuous idiosyncratic disorder seen in a handful of patients.

Blood Brain Barrier (BBB)

It is the most researched portal of entry into the brain in the neuropathology of COVID-19 (neuro-COVID). The components of the BBB are the endothelial cells/BMEC, pericytes, astrocytes, microglia, and neurons. There are three routes into the brain: the olfactory pathway, the breach in the BBB, and the affection of the choroid plexus.

The hypothesized mechanisms of viral entry across the BBB are the trojan horse theory via extracellular “vesicles” and trojan horse trafficking via peripheral immune cells, absorptive transcytosis of viral proteins across BBB (instead of live virus), angiotensin-converting enzyme 2 (ACE2) (also TMPRSS2, NRP1, and PCF) mediated endocytosis of the virus and diffusion of the virus or vesicles containing the viral particles across the tight junction (TJ) without its disruption [6-9]. There is no evidence that live viruses cross the BBB via absorptive transcytosis. However, in one study, the postmortem analysis of the brain by transmission electron microscopy (TEM) shows the presence of viral proteins inside BMECs of the frontal lobe of a patient who died from complications of COVID-19 [9]. However, there were no corresponding pathological changes.

The SARS-CoV-2 is hypothesized to infect the nasal neuro-epithelium and take a retrograde trajectory through the cribriform plate. After that, the virus travels through the olfactory bulb, olfactory nerves, trigeminal ganglion, and trigeminal nucleus in the brainstem in that sequence. Some olfactory nerves also terminate in the amygdala, hypothalamus, and frontal cortex. ACE2 receptors, found on endothelial cells of cerebral arteries, are well-known virus-docking receptors in the CNS. In addition, the TMPRSS2 receptor is frequently co-expressed with ACE2 receptors in the glial cells and is a more efficient portal of entry of SARS-CoV-2. The sustentacular cells in the nasal epithelium express both TMPRSS2 and ACE2 receptors, indicating that these cells may be the viral entrance targets [10]. The direct infection of neuronal cells may not be the initial cause of anosmia but the outcome of viral contact with non-neuronal cells of the mucosal surfaces of the olfactory epithelium and the olfactory bulb [10-13]. Anosmia and ageusia are symptoms of direct viral infection of the neuroepithelium that occur at a rate of 4.96% and 11.35%, respectively [13-15]. The virus has not been experimentally demonstrated to travel on the trajectory in its entirety, and to date, it remains a hypothesis.

The choroid plexus (ChP) lining the ventricles has been proven to show histopathologic changes in COVID-19 patients, such as aberrant cell fusion, tau protein hyperphosphorylation, up-regulation of metabolic processes, cell death, downregulation of tight junctions, and secretory function compromise due to a hypoxic environment [13-16]. The ChP is part of the circumventricular organ (CVO), a designated brain region permeable to factors in the blood. The CVO comprises neurohypophysis, the median eminence, the organum vasculosum of the lamina terminalis (OVLT), the area postrema, the pineal gland, the commissural organ, the sub-fornical organ (SFO), and the ChP [14-17]. The ACE2 receptors are expressed in SFO, ChP, OVLT, pituitary gland, median eminence, area postrema, and periventricular nucleus of the hypothalamus. The TMPRSS2 receptor was predominantly expressed in experimental ChP clusters. Neuropilin-1 (NRP-1), another SARS-CoV-2 receptor, is also expressed in ChP, hippocampal, and cortical organoids [10]. NRP-1 is a multifunctional receptor that mediates angiogenesis dictated by vascular endothelial growth factor (VEGF). The ACE2, NRP-1, and TMPRSS2 receptors act like virus magnets at the ChP. The impaired NRP1 receptor population might cause dysfunctional angiogenesis, thus causing a chronic hypoxic adaptation response in the hippocampus and cortex. As ChP cell damage perpetuates, it could lead to a CSF secretory dysfunction, causing headaches. It explains the prevalence of headaches as one of the most typical neurological complaints. SARS-CoV-2 has also been linked to meningitis, which presumably causes secondary complications such as pseudotumor cerebri or benign intracranial hypertension [15-18]. Future research should be directed towards radio-isotope imaging of tagged astrocytes and microglia to learn whether the initial viral entry into these cells is permissive to subsequent viral neural involvement relevant to recurrent and long-COVID neurological cases.

The most established biomarkers of BBB integrity are the CSF:serum albumin ratio and CSF-albumin concentration since albumin originates exclusively from blood [16-19]. The most frequent CSF pathological finding in 127 COVID-19 patients was elevated blood-CSF barrier QAlb, median 11.4, which was present in 50% of samples from COVID-19 patients without pre-existing CNS diseases. The CSF total protein was elevated in 45.8% of samples, a median of 65.35 milligrams per deciliter, which strongly correlated with QAlb levels. The SARS-CoV-2 CSF polymerase chain reaction (PCR) was negative in all samples. An albumin-cytological dissociation (ACD) was found in 37.4% of samples. The virus-neutralizing CSF-IgG was elevated at 50% but was of a peripheral origin. They found a positive SARS-CoV-2-IgG-antibody index (AI) in two samples. In 56% of samples, pattern-4 oligoclonal bands (OCB) compatible with systemic inflammation were present. Another study on 27 COVID-19 patients with neurological symptoms showed elevated QAlb levels, which correlated with proinflammatory markers such as IL-6, IL-8, and MIP-1 (r=0.6, p <0.01) [19-22]. The two cross-sectional studies demonstrate that systemic inflammation (represented by elevated systemic cytokines and pattern-4 oligoclonal bands) increases BBB permeability (represented by elevated QAlb) and native white cell responsiveness (high SARS-CoV-2-IgG antibody index), foreseeing viral trafficking across BBB.

The monocytic chemoattractant protein-1 (MCP-1) is a pro-inﬂammatory chemokine expressed by neurons, astrocytes, and microglia that is involved in recruiting “inﬂammatory inﬁltrate” into the CNS. Interestingly, one study showed elevated levels of MCP-1 along with normal CSF albumin levels in patients with SARS-CoV-2 encephalopathy, indicating minimal to no BBB leakage. Therefore, suggesting a potential immune cell inﬁltration into the brain without breaching BBB since the albumin was found to be normal [20]. Another study showed that SARS-CoV-2 replicates poorly in cells of human BBB (BMEC and pericytes) [20-23]. However, pericyte and endothelial cells are first to generate a reaction against the virus, and pericyte even initiates the astrocytic release of interferons (IFN-1) which is known to stabilize the BBB [23]. In a study on hamsters and mice, the COVID-19 virus inoculated intranasally was localized using fluorescence in situ hybridization (FISH) in perivascular spaces in the BBB. It was seen abutting the endothelial cells of BBB. In the same study, they visualized virus-like particles ultrastructurally inside pericytes. This cerebrovascular endotheliopathy is typical of CNS-tropic viruses and can be transient or chronic in cases of SARS-CoV-2. In the latter case, it leads to symptoms of long-COVID [23-25].

Neurofilament light chain (NfL) is specific for neuronal damage, the increased plasma NfL levels in patients without neurological symptoms suggest the presence of subclinical CNS involvement in patients with a history of COVID-19, especially in those with the most severe forms of the disease. In one study, COVID-19 patients who developed acute respiratory distress syndrome (ARDS) during the acute phase of the disease tended to maintain higher levels of NfL even up to three months after hospital discharge. Another study on CSF and plasma NfL and CSF MMP-2 levels showed significantly higher CSF and plasma NfL and CSF MMP-2 levels in ARDS than in the non-ARDS group. Plasma NfL is moderately related to CSF NfL. SARS-CoV-2 RNA detection was not associated with increased CSF NfL and MMP-2 levels. Another study showed that there is a steep decline in NfL levels in post-acute sequelae of SARS-CoV-2 patients. Para-infectious antibodies might form in a time spanning from the peak of NfL levels until immunological homeostasis is reached, which leads to post-acute sequelae [25-28]. Live SARS-CoV-2 has never been isolated from an acute neurological case and it is not confirmed that the virus crosses the BBB to cause disease.

On the contrary, the acute phase of CNS damage in COVID-19 patients is related to respiratory COVID-19 severity rather than SARS-CoV-2 neuro-invasion. Viral particle trafficking across BBB remains a stochastic process, and BBB disruption is not a pre-requisite for viral entrance but a corollary of the virus’ endeavours at entry into CNS and secondary to BBB dysfunction due to systemic causes. A study to discover cytokines and interleukins associated with SARS-CoV-2 discovered CCL7, CCL2, MCP1, interleukin-32 (IL-32), IL-18, and IL-8 generated by ChP organoid clusters infected with SARS-CoV-2 [3-4]. The cortical microglia undergo transcriptional modifications that characterize augmented microglia activity, motility, and phagocytosis in COVID-19. It is not consistent with a breakdown of the BBB but with the activation of immune cells native to CNS. The glial and neural cell activation markers, such as CSF neopterin, beta-2 microglobulin, sTREM2 (soluble ectodomain of triggering receptor expressed on myeloid cells 2), and GFAp (glial fibrillary acidic protein), were found to be significantly elevated in CSF studies of neurological COVID-19 patients. Potentially prognostic, these biomarkers are directly proportional to the CSF albumin levels signifying that BBB leakage occurs proportional to the severity of neurological involvement in COVID-19 [25-30].

A previously compromised endothelial barrier function in hypertension, diabetes, and obesity may increase pericyte exposure and promote virus-pericyte interaction [29]. The local inflammation subsequent to vascular infection may explain in part why these vascular risk factors are associated with severe COVID-19, as they may also contribute to thromboembolic events [29-33].

Brain

The most prevalent neurological symptoms of COVID-19 are headaches (1.7-33.9%), dizziness, altered consciousness, acute cerebrovascular insufficiency, amnesia, seizures (1.6% in 4491 COVID-19 patients), and ataxia [30-34]. Thirty men and seven women with COVID-ARDS, spanning an age range from 60-80 years, were included in a study, and only one patient tested positive for SARS-CoV-2 RNA in the CSF. The most prevalent neurological manifestations were altered consciousness (73%), pathological wakefulness upon cessation of sedation (41%), confusion (32%), and agitation (19%). The medial temporal lobe hyperintensities were seen in 43% of patients.

Encephalitis is reported in a lower frequency of 0.22% than encephalopathy, which occurred at a rate of 8.7% amongst 12,601 hospitalized COVID-19 patients and affected patients with severe COVID-19 infection more than others. CSF pleocytosis, MRI abnormalities, and electroencephalographic (EEG) change often characterize encephalitis [32-36]. The intrathecal IgG production, seen in some SARS-CoV-2-induced encephalitis cases, is a virus-neutralizing immune response that reduces viral trafficking across BBB. COVID-19-related encephalopathy often does not present with CSF pleocytosis or MRI abnormalities and seems reversible [35-38]. Patients with COVID-19 can develop a corticosteroid-responsive encephalopathy involving any part of the brain [38]. Encephalopathy appears to have a favourable prognosis compared to encephalitis in SARS-CoV-2 [38-40]. Other well-defined cerebral neurological diseases caused by SARS-CoV-2 include acute disseminated encephalomyelitis (ADEM) and acute hemorrhagic necrotizing encephalitis. The ADEM phenotype seen in SARS-CoV-2 has oculomotor dysfunction, seizures, delirium, and coma. The neuroimaging abnormalities in neuro-COVID include stroke, leptomeningeal enhancements, isolated white mater microhemorrhages, cytotoxic lesions of the corpus callosum, hyperintense lesions in the mesial temporal lobes, medial thalami, claustrum as well as white matter (multifocal and non-confluent) on T2 weighted and fluid-attenuated inversion recovery (FLAIR) sequences [34-43]. The cytotoxic lesions of the corpus callosum are para-infectious. COVID-19 is associated with an increased incidence of large vessel stroke [40-47]. The ischemic stroke rate was 1.2% in 4466 COVID-19 patients [44-50]. Other forms of stroke related to SARS-CoV-2 include cerebral venous thrombosis (CVT), branch vessel stroke, lacunar stroke, cryptogenic, free-floating thrombus, and watershed zone strokes. An intracerebral haemorrhage is a complication of long-term hospitalization or a long course of the disease, with a rate of 0.46% in 67,155 COVID-19 patients, and it is exacerbated by non-judicious anticoagulant use [30-50]. COVID-19 meningoencephalitis is characterized by punctate or diffuse T2 and FLAIR hyperintensities in the subcortical white matter, brainstem, temporal lobe, thalami, and claustrum. Cerebral edema often accompanies these findings [45-50]. A symmetric bi-hemispheric involvement indicates systemic inflammatory spillover rather than local pathology.

The multisystem inflammatory syndrome (MIS) seen in COVID-19 presents as fever with rash, thrombocytopenia, hypovolemic shock, and GI symptoms. The neurological manifestations are headache with vomiting, confusion, drowsiness, and stupor. It occurs three to six weeks post COVID-19 infection and COVID-19 vaccination [45-50]. It is commoner in children than adults. The laboratory findings include elevated procalcitonin, C-reactive protein (CRP), and erythrocyte sedimentation rate (ESR) (CDC).

Posterior reversible encephalopathy syndrome (PRES), a condition of disrupted cerebrovascular autoregulation and endothelial dysfunction causing preferential hyperperfusion of the posterior circulation, has also been reported as a neurological consequence of COVID-19, a cause obscured by concurrent dialysis [45-55]. In one study, a brain positron emission tomography (PET) scan using 18-FDG demonstrated putaminal and cerebellar hypermetabolism in conjunction with widespread cortical hypometabolism, as validated by whole-brain voxel-based statistical parametric mapping (SPM) quantification, congruent with the diagnosis of encephalitis and cerebellitis. A T2-weighted and FLAIR axial image of the putamen and cerebellum revealed no abnormalities. A high titer of IgG autoantibodies targeting the nuclei of Purkinje cells, striatal neurons, and hippocampal neurons was detected in serum and CSF by the immunologic assay of the same patient. Steroid treatment permitted a rapid improvement in symptoms indicating a postinfectious aetiology. Other acute neurological syndromes in SARS-CoV-2 are diffuse hypoxic damage and post-hypoxic demyelination.

A case of optic neuritis one week after the onset of COVID-19 illness was reported in a 16-year-old who complained of a painful loss of vision in one eye. In addition, there was disc edema on indirect ophthalmoscopy, prolonged P100 latency on visual evoked potentials (VEP), positive relative afferent pupillary defect (RAPD), and a hyperintense optic nerve on MRI orbital nerve sequence. The pathogenesis of optic neuritis appeared to be parainfectious, all other causes having been ruled out. Optic neuritis can occur alone or in conjunction with other COVID-19-induced neurological illnesses, including myelitis, neuromyelitis optica (NMO) spectrum disorders, anti-myelin oligodendrocyte glycoprotein (anti-MOG) antibody disease, and acute disseminated encephalomyelitis (ADEM) [49-55].

Parkinsonism, cognitive impairment, mental dullness, anxiety, and depression are long-term neurological complications seen in COVID-19.

Brainstem and Spinal Cord

The highest expression of ACE2 was found in the amygdala, pons, and medulla oblongata [52]. The latter two regions contain the respiratory control centers of the brainstem. As it was predicted that the varied neurological manifestations of COVID-19 are connected with the degree of ACE2 receptor expression in particular brain regions, the brainstem was designated the CNS viral hotspot. Across all studied viruses, brain organoids have mainly been used to investigate the susceptibility of the CNS to viral infection (90% of studies), and viral tropism (54% of studies) [50-55]. ACE2 receptors were disproved to be an efficient mode of entry across the BBB as SARS-CoV-2 failed to show the difference in permeability across a human BBB organoid compared to control under experimental conditions. SARS-CoV-2, long thought to affect the pre-Botzinger complex in the medulla, has been equivocally identified in “live” form in the brainstem in postmortem studies and human brainstem organoids.

Conversely, one author reported hyperintensities in the midbrain, pons, and medullary reticular formation, which were associated with a high respiratory rate, between 40 and 45 breaths per minute in a patient. The author did not give plethysmographic information to assess the type of breathing pattern in the article. The strong association between active pulmonary COVID-19 and hyper-intense respiratory centers suggests brainstem encephalitis rather than direct viral invasion.

Facial diplegia, lateral geniculate body syndrome, Landry-GBS (LGBS), polyneuritis cranialis with ataxia, brainstem encephalitis, brainstem stroke, and brainstem (or spinal cord) myelitis are all described in association with SARS-CoV-2. In addition, GBS in COVID-19 often manifests during the first two weeks of the illness, suggesting that there may be an overlap between active SARS-CoV-2 infection and the development of GBS, which supports a more para-infectious than postinfectious pathogenesis [35-40,50-55].

Myelitis, the inflammation of the spinal cord, can have various etiologies, including autoimmune, viral infection, and even paraneoplastic, a sampling of the items on the endless list of causes. When accompanied by SARS-CoV-2, myelitis can adversely impact any spinal cord level. Transverse myelitis caused by SARS-CoV-2 may damage a short spinal cord segment (three or fewer vertebrae) or affect a large portion of the spinal cord longitudinally (more than three vertebral levels of the spinal cord). According to a case series, 75% of patients had a history of COVID-19 disease at least two weeks before developing acute onset transverse myelitis. These had negative nasopharyngeal reverse transcription (RT)-PCR for COVID-19 and no active COVID-19 illness at the time of paralysis, but COVID-19 antibodies were positive. In the remaining 25% of the individuals, nasopharyngeal RT-PCR was positive, and the COVID-19 illness was contemporaneous; antibodies were negative. Each patient complained of limb weakness as well as alterations in the bowel and bladder. A sensory level was present in only one case of longitudinally extensive transverse myelitis starting at T2 who presented with quadriparesis [50-60].

Cerebellum

Cerebellar syndrome can present as acute cerebellar dysfunction due to postinfectious or parainfectious cerebellitis. The common cerebellar manifestations of SARS-CoV-2 are tremor, ataxia, dysarthria (23% in 315 COVID-19 patients), cerebellar stroke, and opsoclonus-myoclonus syndrome (63.4%, in 59 COVID-19 patients), upper-limb dysmetria associated with spontaneous diffuse myoclonus, which affects the proximal limbs, worsens with movement, and are stimulus-sensitive [55-60]. The neurobehavioral syndrome (paranoia, irritability, aggression, and disinhibition) is thought to be caused by acute lesions of the cerebellar posterior lobe and vermis, which disrupt cortico-cerebellar pathways and involves the prefrontal, posterior parietal, temporal, and limbic cortex, and invites for multidisciplinary input from psychiatry and intensive neurorehabilitation. In a similar case report, the axial T2-weighted MRIs revealed aberrant signal intensities in the left thalamus, bilateral occipital lobes, cerebellar vermis, and left cerebellar hemisphere secondary to posterior circulation infarctions. Human postmortem studies have shown the occurrence of neuronophagia and bilateral multifocal microglial nodules in the inferior olives and cerebellar dentate nuclei without clinical correlates [55-60].

A case of quadriparesis was reported with no visual manifestations. An MRI revealed hyperintense lesions in cerebellar peduncles and pons. The IgG anti-MOG antibodies were strongly positive, and the anti-spike protein COVID-19 antibodies were increased. A diagnosis of MOG disease was made. A recent history of the administration of the COVID-19 vaccination suggested the risk of MOG antibody-associated disease (MOGAD) related to immunization [58-65].

ANS and COVID-19

Heart rate variability (HRV) is a known feature reported in cross-sectional studies on hospitalized COVID-19 patients. Higher HRV predicts better survival, notably in COVID-19 patients aged 70 and older, and is independent of other critical prognostic parameters. Conversely, in the first week after hospitalization, a low HRV predicts ICU admission [60-65].

Despite its recognition, phrenic nerve involvement in neuralgic amyotrophy is uncommon. An ultrasound study of the phrenic nerve in a few COVID-19 patients discovered reduced phrenic nerve thickness and hypoechogenicity of the phrenic nerve and distal diaphragmatic tissue atrophy [55-64]. SARS-CoV-2 has also been linked to diaphragmatic myoclonus. EEG recordings revealed lateralized periodic discharges (LPDs) that were synchronous and asynchronous with diaphragmatic myoclonic movements. However, an association of the singultus to distinct CNS foci could not be established. There have also been three reports of widespread myoclonus caused by SARS-CoV-2 infection [60-68]. 

PNS and COVID-19

A case series identified three distinct types of GBS in three COVID-19 patients: (i) the garden variety (demyelinated sensorimotor polyradiculoneuropathy), (ii) The acute motor axonal neuropathy (AMAN) variety (pure motor neuronal axonopathy), and (iii) the Miller Fisher variant of GBS (impersistent F waves). After contracting COVID-19 and getting cured, each patient manifested GBS symptoms within two to four weeks and had a rate of 0.15% among the 136,746 COVID-19 patients [65]. Similar to non-COVID-19 GBS cases, the weakness began in all patients’ lower limbs and progressed to the upper limbs. They all had IVIG treatment and recovered completely [60-68].

The autoimmunity in COVID-19-associated GBS is attributed to anti-ganglioside antibodies that cause the autoimmune polyradiculoneuropathies, such as those caused by anti-GQ1b, anti-GM1164, and anti-GD1b antibodies. They have been detected in patients with COVID-19 presenting with areflexic weakness, cranial neuropathies, and sensory ataxia. Compared to acute inflammatory demyelinating polyneuropathy (AIDP), anti-ganglioside antibodies are more strongly linked to aggressive axonal motor neuropathies and lower functional results. The rarity of these antibodies echoes with molecular mimicry generated by the SARS-CoV-2 vaccine, which could trigger autoimmune responses raising concerns for vaccine safety [60-68]. In addition, some authors described the presence of leptomeningeal enhancement, which is an atypical feature of GBS, as a marker of GBS's association with SARS-CoV-2 infection [65-68].

In cases of Miller Fisher syndrome (MFS) caused by COVID-19, the most notable symptoms included perioral paresthesias, ataxia, blurred vision, ophthalmoplegia, generalized areflexia, and weakness. Although anti-GQ1b antibodies and other anti-ganglioside antibodies are negative, the presentation period of MFS concerning COVID-19 illness suggests a postinfectious immune activation. Similar scientific literature shows that a COVID-19-associated MFS case has a distinct immune mechanism or antigen target than non-COVID-19 MFS. Conversely, Bickerstaff brainstem encephalitis, also part of the anti-GQ1b IgG antibody syndrome spectrum like MFS, is described in conjunction with pediatric SARS-CoV-2 infection, pointing at the abundance of this antigen in the nervous system. It has been hypothesized that NfL may be used to detect early involvement of the nervous system in COVID-19-associated MFS patients [63].

However, neuropathy and myopathy are more prevalent than GBS. For example, one study reported a combined rate of 56.3% among 400 hospitalized COVID-19 patients [67]. The rate of neuropathic pain can be as high as 2.3% in hospitalized patients with COVID-19. In addition, some authors have described neuropathic pain in SARS-CoV-2 concerning neuralgic amyotrophy, brachial plexopathy, and small fiber neuropathy [65-68]. However, Bell’s palsy is the most common isolated PNS disorder in neuro-COVID, with an incidence rate of 0.08% [66-68].

Neuromuscular junction

Myasthenia gravis (MG) is rare in COVID-19 patients. Myasthenic crises, respiratory failure, and mortality from cytokine storms are more likely to occur in MG patients infected with COVID-19. Therefore, patients diagnosed with MG due to COVID-19 should be managed in a multidisciplinary critical care unit [60-68].

Musculoskeletal system

Myalgic encephalitis syndrome or chronic fatigue syndrome (CFS) is a long-term neurological complication characterized by exercise intolerance, cognitive changes, altered sleep stages, and the need for prolonged rest. The prevalence of CFS is as high as 45.2%, as found in an epidemiological study done on 127,117 COVID-19 patients. Seven-Tesla MRI studies have revealed an overactive metabolism in the brain with a decrease in GABA in the anterior cingulate cortex and an increase in glutamate and glutamine in the putamen. In addition, some researchers report an elevation in intraventricular lactate due to hypoxia, which may be the result of anaerobic metabolism [68].

## Conclusions

The SARS-CoV-2 virus RNA has been identified in a spectrum of brain regions with no discernible histopathological markers of infection. On the contrary, CNS inflammation (particularly in the olfactory bulb and brainstem), accompanied by microvascular damage and microglial activation, was observed in the absence of in vivo virions or virus-infected cells. The SARS-CoV-2 viral genomes and proteins can be detected in the brain tissue of diseased patients, although there is a paucity of neuropathologic manifestations of infection in these areas. In rare instances, viral RNA can be detected in the CSF of patients with neurological clinical features. However, most patients with neurological symptoms do not have evidence of active viral replication in the nervous system. Typical clinical and laboratory features of viral CNS infections are infrequent. Endotheliopathy and the systemic inflammatory response to SARS-CoV-2 infection play a pivotal role in the development of cerebral COVID, with glial cell activation due to proinflammatory cytokine playing a vital role in both processes. The inflammatory mediators presumably operate across the BBB to generate neuroinflammation by activating astrocytes and microglia, resulting in indirect neuroinflammation and nervous system damage. Most subacute neurological disorders have a parainfectious etiology. Depending on the length of time from the COVID-19 infection, the first line of treatment for neurological disorders may involve either plasmapheresis (or IVIG) or intravenous steroids.
